# Enhanced sentiment analysis in tourism reviews via multimodal graph convolutional networks

**DOI:** 10.1371/journal.pone.0333628

**Published:** 2025-12-29

**Authors:** Weijia Yang, Zilian Xue, Guitao Fu, Guodong Chen, Cunyuan Yang

**Affiliations:** 1 School of Art and Design, Zhejiang Agriculture and Forestry University, Hangzhou, Zhejiang Province, China; 2 Faculty of Engineering, University of Sydney, Sydney, Australia; 3 School of Art and Design, Zhejiang Agriculture and Forestry University, Hangzhou, Zhejiang Province, China; 4 School of Art and Design, Zhejiang Agriculture and Forestry University, Hangzhou, Zhejiang Province, China; 5 School of Creative Design, Zhejiang Mechanical and Electrical Vocational and Technical, Hangzhou, Zhejiang Province, China; PLOS: Public Library of Science, UNITED KINGDOM OF GREAT BRITAIN AND NORTHERN IRELAND

## Abstract

In recent years, multimodal sentiment analysis has gained prominence due to its ability to leverage diverse data types for improved accuracy. However, combining text and image modalities presents challenges in effectively integrating and processing these diverse data sources for more effectively sentiment analysis. This study introduces a novel multimodal graph convolutional network GraphSAGE sentiment analysis model that integrates text and image features using advanced techniques include BERT for text graph construction and ResNet for image feature extraction and image graph construction, combined with a multi-head attention mechanism for effective edges fusion and nodes fusion. Experimental results on several datasets demonstrate that the proposed model outperforms existing state-of-the-art methods in terms of accuracy and efficiency. The findings highlight that the proposed model using multimodal data significantly enhance sentiment analysis performance. The source code for this study can be viewed at this url: https://github.com/CunyuanYang/Muilti-model_ESA.

## Introduction

In recent years, with the rapid development of the internet and social media, the amount of user-generated content [1] on online platforms has grown exponentially. This content includes vast amounts of multimodal data, such as text, images, and audio, reflecting users’ emotions and opinions [2,3]. Therefore, extracting valuable information, particularly sentiment information, from this multimodal data has become a significant research topic. Sentiment analysis, which involves automatically identifying and extracting emotional states from text, images, audio, and other data, is a core task at the intersection of natural language processing, computer vision, and multimodal computing [4–8].

Traditional sentiment analysis methods primarily focus on single-modality data, such as analyzing text [9] or images [10] alone. However, single modality approaches often fail to capture the complexity of users’ emotions comprehensively [11]. For instance, while text may convey a certain sentiment, accompanying images or audio might convey subtler or even contradictory emotional information. Therefore, combining multimodal data for sentiment analysis is crucial, as it can enhance the accuracy and robustness of sentiment detection.

Multimodal Graph Convolutional Neural Networks (MGNNs) represent an emerging deep learning method that offers an effective means of integrating and processing multimodal data [12–14]. MGNNs utilize graph structures to represent different modalities’ data nodes and their relationships, leveraging graph convolution operations to capture complex dependencies both within and across modalities. This approach not only effectively utilizes the unique information of each modality but also achieves more comprehensive and accurate sentiment recognition results through multimodal fusion. Sentiment analysis based on MGNNs has shown tremendous potential in various domains, including social media analysis, recommendation systems, and medical image analysis. By effectively integrating and analyzing multimodal data, MGNNs help us better understand users’ emotions and needs, thereby promoting the development and application of intelligent systems. The ability to accurately gauge sentiment through multimodal data fusion can lead to more nuanced insights, ultimately improving user interaction, content recommendation, and personalized services across diverse platforms. The application of MGNNs to sentiment analysis represents a significant advancement in the field, addressing the limitations of single-modality approaches and leveraging the rich, complementary information available across different data types. This research aims to explore the design and implementation of MGNN-based sentiment analysis methods, demonstrating their efficacy and highlighting the broader implications for the future of multimodal data analysis in various practical applications. In current sentiment analysis research, the noise and complexity of data in real-world datasets make it insufficient for single-modal information to comprehensively capture users’ emotions. Moreover, existing multimodal sentiment analysis methods often struggle with effectively integrating textual and visual features.

This study proposes a multimodal GCN sentiment analysis model. The design of this multimodal sentiment analysis model integrates various deep learning techniques include BERT, ResNet [15] and GraphSAGE to enhance sentiment prediction accuracy. Firstly, BERT is used for text encoding to construct a text graph (text-Graph), capturing rich contextual information from textual data. Concurrently, ResNet is employed to extract features from images, creating an image graph to represent visual content and the relationships between it. To fuse these two modalities, the proposed model utilizes a multi-head attention mechanism, which performs weighted fusion of the nodes and edges of both the text graph (text-Graph) and the image graph (image-Graph). This fusion process ensures that the model can effectively leverage complementary information from both text and images. Subsequently, the model employs the GraphSAGE algorithm to train the multimodal graph convolutional network, enabling the model to learn and aggregate information from the multimodal graph structure. To optimize model performance, two loss functions are proposed: one for maximizing sentiment prediction accuracy and another for ensuring balance among different sentiment categories. These loss functions are combined into a total loss function through weighted summation, facilitating a balanced and effective training process.

This model offers several advantages: it leverages the strengths of BERT and ResNet for high-quality feature extraction, employs a multi-head attention mechanism to effectively integrate multimodal data with diverse emotional information, and applies the GraphSAGE algorithm to handle complex graph structures. The dual loss functions ensure prediction accuracy and class balance, addressing common issues of class imbalance in sentiment analysis and enhancing the overall robustness of the model.

The four innovations of this study can be summarized as follows:

1. Integration of BERT and ResNet for Multimodal Graph Construction: This study uniquely combines BERT for text encoding and ResNet for image feature extraction to construct separate text and image graphs.
2. Application of Multi-Head Attention Mechanism for Multimodal Fusion: The study introduces a multi-head attention mechanism to weight and integrate the nodes and edges of the text graph and image graph.
3. Dual Loss Functions for Balanced Optimization: The study combines two distinct loss functions—one for maximizing sentiment prediction accuracy and another for ensuring class balance.
4. Introduces GraphSAGE as an innovative approach, leveraging its efficient neighbor sampling and feature aggregation mechanism to address the computational bottlenecks of traditional graph convolutional networks when handling large-scale graph data.

In the rest of this paper, we will introduce the recent related work in section 2. Section 3 presents the proposed methods: overview, overview of the proposed model, data collection and preprocessing, construction of text graph, construction of image graph, multimodal graph fusion algorithm, sentiment analysis based on multimodal graph convolutional neural networks. Section 4 introduces the experimental part, including practical details, comparative experiments, and an ablation study. Section 5 includes a conclusion.

## Related work

### Online platform sentiment analysis

Sentiment analysis [16], also known as opinion mining, is a subfield of natural language processing (NLP) that focuses on identifying and extracting subjective information from text data. Its purpose is to determine the sentiment expressed in a piece of text, which can be classified as positive, negative, or neutral. The core principles of sentiment analysis involve processing text data to understand users’ emotions, opinions, and attitudes. This process typically includes steps such as text preprocessing, feature extraction, sentiment classification, and result interpretation.

The literature on online platform sentiment analysis covers a wide range of topics and methodologies. Paper [17] propose a system that utilizes factor aggregation of sentiment polarization to analyze hotel reviews and predict hotel quality. Paper [18] discuss the methodology of determining public opinions through sentiment analysis design using Python data mining. Paper [19] introduce deep learning NLP models for sentiment analysis and product review classification in e-commerce platforms. Paper [20] examine the correlation between user review features and their usefulness in the context of online mental health consultation services. Paper [21] apply topic modeling and sentiment analysis to explore user perceptions of an online learning platform during the COVID-19 pandemic. Overall, these studies demonstrate the diverse applications of sentiment analysis in various online platforms, from e-commerce to mental health consultation services, and the importance of understanding user sentiment for improving services and predicting platform failure risks.

However, the accuracy of sentiment analysis models heavily depends on the quality of the input data. Noisy, unstructured, or imbalanced datasets can degrade model performance. Effective data preprocessing and augmentation techniques are crucial but can be time-consuming. Moreover, despite the high accuracy of many deep learning models, they often operate as black boxes. Their complex architectures make it difficult to interpret how they arrive at specific predictions, which can be a limitation in applications requiring transparency. Additionally, sentiment analysis models trained in one domain (e.g., movie reviews) may not perform well in another domain (e.g., product reviews) due to differences in language use and context. Developing models that can generalize across domains remains a significant challenge. Finally, training sophisticated models like BERT requires substantial computational resources and time, which can be a barrier for smaller organizations or researchers with limited access to high-performance computing infrastructure.

### Multimodal sentiment analysis

Multimodal sentiment analysis [22–26] is an advanced subfield of sentiment analysis that enhances the accuracy and robustness of sentiment classification by integrating multiple types of data, such as text, images, audio, and video. The core principle behind multimodal sentiment analysis is that different data modalities provide complementary information that, when combined, offer a more comprehensive understanding of sentiment. For example, textual data can provide context and specific sentiment cues, while visual data can convey emotions through facial expressions or scenes, and audio data can capture tone and intonation in speech.

The process of multimodal sentiment analysis typically includes the following steps:

***Data Collection:*** Gathering data from various sources and ensuring that the data from different modalities are aligned.

***Preprocessing:*** Cleaning and preparing data for analysis, including normalization, segmentation, and feature extraction specific to each modality.

***Feature Extraction:*** Using models such as BERT for text feature extraction, CNN for image feature extraction, and LSTM for audio feature extraction.

***Fusion Techniques:*** Combining features from different modalities using methods like concatenation, attention mechanisms, or more complex approaches such as tensor fusion.

***Classification:*** Employing machine learning or deep learning models to classify sentiment based on the fused features.

***Post-processing and Interpretation:*** Analyzing the results and providing interpretations that can be used for decision-making or further research.

Multimodal sentiment analysis is a burgeoning field that aims to leverage multiple modalities such as text, audio, and video to understand and predict sentiment and emotions in various contexts. Researchers have proposed various innovative frameworks and models to address the complexities of analyzing sentiment across different modalities. Paper [27] introduced a quantum-like multimodal network (QMN) that combines quantum theory formalism with LSTM networks to model interaction dynamics in multiparty conversational sentiment analysis. This framework aims to accurately capture the intricate interactions that occur during conversations. Paper [28] proposed the Interaction Canonical Correlation Network (ICCN) to learn hidden correlations between text, audio, and video features for improved multimodal sentiment analysis and emotion recognition. By exploring relationships between different modalities, this model enhances the understanding of sentiment expressed through various channels. Paper [29] focused on learning modality-invariant and -specific representations for multimodal sentiment analysis to facilitate the fusion process. Their work emphasizes the importance of effective modality representations in enhancing the overall analysis of user-generated videos. Paper [30] introduced the Multimodal Adaptation Gate (MAG) attachment to BERT and XLNet for integrating multimodal information in large pretrained transformers. Their experiments on CMU-MOSI and CMU-MOSEI datasets showcase the potential of leveraging multimodal data for sentiment analysis. Paper [31] proposed TransModality, an end-to-end fusion method with Transformer, to predict speaker sentiment tendencies using textual, visual, and acoustic modalities. Inspired by the success of Transformers in machine translation, this method offers a new approach to multimodal sentiment analysis. These studies highlight the importance of effective fusion methods, modality representations, and dynamic modeling for enhancing the accuracy and comprehensiveness of sentiment analysis in multimodal data. Table 1 gives the detailed comparison of these works.

**Table 1:** Comparative analysis of work related to multimodal sentiment analysis.

Citation	Model Name	Fusion Strategy	Modality Processing	Core Innovation	Limitations
[27]	Quantum-like Multimodal Network (QMN)	Quantum-theoretic fusion (interaction dynamics modeling)	Text, Audio, Video → Quantum state representations	Combines quantum theory with LSTM to model multiparty conversational dynamics	High computational complexity: theoretical abstraction may limit interpretability
[28]	Interaction Canonical Correlation Network (ICCN)	Statistical correlation learning (hidden cross-modal correlations)	Text, Audio, Video → Canonical correlation analysis	Learns latent correlations between modalities via canonical correlation	Assumes modality alignment; struggles with unaligned data
[29]	MISA (Modality-Invariant/Specific Representations)	Feature disentanglement (modality-invariant + modality-specific features)	Text, Video → Adversarial learning + similarity constraints	Separates shared and unique modality features for flexible fusion	Requires careful balance between invariance/specificity losses
[30]	Multimodal Adaptation Gate (MAG)	Gating mechanism (BERT/XLNet adaptation)	Text, Audio, Video → Modality-specific gates attached to transformers	Enhances pretrained transformers (BERT/XLNet) with lightweight multimodal gates	Limited to transformer-based architectures; gate design may oversimplify interactions
[31]	TransModality	End-to-end cross-modal attention (Transformer-based)	Text, Visual, Acoustic → Unified transformer encoder	Extends Transformer architecture for joint multimodal encoding-decoding	High parameter count; requires large-scale training data

However, one of the primary challenges in multimodal sentiment analysis is ensuring that data from different modalities are properly aligned and synchronized. Misalignment can lead to incorrect sentiment predictions and reduced model performance. Additionally, multimodal models are inherently more complex and computationally intensive than unimodal models. They require substantial computational resources for training and inference, which can be a barrier for smaller organizations. Furthermore, high-quality multimodal datasets are relatively scarce, and existing datasets often suffer from class imbalance, where certain sentiments or modalities are underrepresented. This imbalance can negatively impact the model’s ability to generalize. While multimodal models can achieve high accuracy, their complexity often makes them less interpretable. Understanding how different modalities contribute to the final prediction can be challenging, especially in applications requiring transparency.

### Multimodal graph convolutional neural network

Multimodal Graph Convolutional Neural Networks (MGCNs) are advanced extensions of Graph Convolutional Networks (GCNs), designed to process and integrate multiple data modalities (such as text, images, and audio) into a unified graph-based framework. The core principle of MGCNs is to leverage the complementary information provided by different data types and capture their interrelationships through a graph structure. Each modality is represented as a graph, where nodes correspond to data entities (such as words, image regions, or audio frames), and edges represent the relationships or interactions between these entities. By integrating these modality-specific graphs into a single multimodal graph, MGCNs can directly perform convolutions on the graph, aggregating features from neighboring nodes and capturing the intrinsic features and structural information within the data.

The literature on multimodal graph convolutional neural networks (GCNs) has seen significant advancements in recent years. Paper [32] introduced a deep model for cloud classification using heterogeneous deep features (HDFs) extracted through a convolutional neural network (CNN) that combines visual and multimodal information. Paper [33] explored the effectiveness of graph neural networks (GNN) in analyzing unaligned multimodal sequences with their Multimodal Graph model. Paper [34] proposed a self-attention enhanced spatial-temporal graph convolutional network for skeleton-based emotion recognition, incorporating pose estimation for 3D skeleton coordinates. Paper [35] developed a graph-based deep neural network to model brain structure and function in Mild Cognitive Impairment (MCI), resulting in a Deep Brain Connectome that maximizes the capability of differentiating MCI patients from normal controls. Paper [36] presented a TechDoc architecture that leverages natural language texts, descriptive images, and document associations for technical document classification using a combination of CNN, RNN, and GNN. These studies collectively demonstrate the diverse applications and advancements in multimodal graph convolutional neural networks across various domains.

One of the main challenges in implementing MGCNs is ensuring the correct alignment and synchronization of different modalities. Misalignment can lead to incorrect feature aggregation and reduced model performance. Additionally, compared to unimodal GCNs, MGCNs are more complex and computationally intensive. Their training and inference require substantial computational resources, which can be a barrier for small organizations.

## Method

### Overview of the proposed model

**Fig 1:**
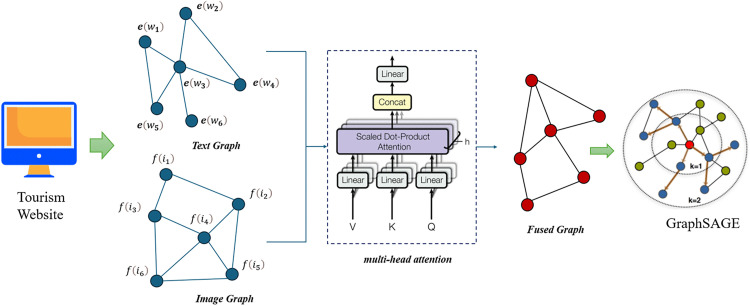
The proposed multimodal sentiment analysis model.

This comprehensive architecture effectively captures and integrates multimodal information, leveraging the strengths of BERT, ResNet, multi-head attention, and GraphSAGE to deliver robust sentiment analysis. The structure of the proposed model is shown in Fig 1.

The proposed multimodal sentiment analysis model leverages a combination of advanced deep learning techniques to integrate textual and visual information effectively. The model’s architecture consists of several key components, each designed to capture and fuse features from different data modalities, ultimately enhancing sentiment prediction accuracy.

1 Text Encoding using BERT: The textual data is processed using the Bidirectional Encoder Representations from Transformers (BERT). Let $T=\{t_{\text{1}},t_{\text{2}},\ldots ,t_{n}\}$ represent the sequence of tokens in the text. BERT encodes this sequence into contextual embeddings $E_{T}=\{e_{\text{1}},e_{\text{2}},\ldots ,e_{n}\}$, where $e_{i}\in R^{d}$.

$$E_{T}=\text{BERT}(T)$$ (1)

2 Text Graph Construction: A text graph $G_{T}=\left(V_{T},E_{T}\right)$ is constructed, where nodes $V_{T}$ correspond to the token embeddings, and edges $E_{T}$ represent the syntactic and semantic relationships between tokens.
3 Image Feature Extraction using ResNet: Visual data is processed using a Residual Network (ResNet). Given an image I, ResNet extracts a feature map $F_{I}$.

$$F_{I}=\text{ResNet}(I)$$ (2)

4 Image Graph Construction: An image graph $G_{I}=\left(V_{I},E_{I}\right)$ is constructed, where nodes $V_{I}$ represent image regions described by the feature map $F_{I}$, and edges $E_{I}$ capture spatial relationships between these regions.
5 Multimodal Graph Fusion using Multi-Head Attention: To integrate text and image graphs, a multi-head attention mechanism is applied. Let $H_{T}$ and $H_{I}$ be the feature matrices for the text and image graphs, respectively. The attention mechanism computes the fused representation $H_{F}$.

$$H_{F}=\text{MultiHeadAttention}\left(H_{T},H_{I}\right)$$ (3)

6 Graph Convolution using GraphSAGE: The fused graph $G_{F}=\left(V_{F},E_{F}\right)$, with nodes $V_{F}$ and edges $E_{F}$, undergoes convolution using the GraphSAGE algorithm. The node embeddings $Z_{F}$ are updated through aggregation functions.

$$Z_{v}=\sigma \left(W\cdot \text{AGGREGATE}\left(\{h_{u},\forall u\in N(v)\}\right)\right),\;\forall v\in V_{F}$$ (4)

where W is the parameter to be trained.

7 Dual Loss Function Optimization: Two loss functions are proposed: $L_{\text{acc}}$ for maximizing sentiment prediction accuracy and $L_{balance}$ for ensuring class balance. The total loss L is a weighted sum of these two functions.

$$L=\alpha \cdot L_{\text{acc}}+\beta \cdot L_{balance}$$ (5)

where α and β are the parameters that adjust the weights of the two loss functions.

For text feature extraction, BERT was selected over alternatives like Transformer, GPT, or T5 due to its bidirectional context capture capability through masked language modeling, which is particularly effective for analyzing sentiment-laden tourism reviews where contextual understanding of both preceding and succeeding words is crucial. Unlike GPT’s unidirectional approach or T5’s text-to-text framework, BERT’s pretrained representations on large corpora provide superior semantic understanding of review texts.

Regarding image processing, ResNet’s residual connections address vanishing gradient problems in deep networks, making it more effective than plain CNN architectures (e.g., VGG) for extracting hierarchical visual features from tourism images where objects may appear at varying scales and perspectives. We specifically chose ResNet-50 as it offers an optimal balance between computational efficiency and feature extraction performance compared to shallower or deeper variants. These architectural advantages make BERT and ResNet particularly suitable for the proposed multimodal graph construction where robust feature extraction from both modalities is essential for subsequent fusion and sentiment analysis. The modifications have been integrated into the relevant paragraphs while maintaining all existing technical descriptions of the implementation.

GraphSAGE was specifically chosen due to its inductive learning capability through neighbor sampling, which enables efficient processing of large-scale tourism review graphs that may contain dynamic or previously unseen nodes – a critical advantage over transudative methods like GCN that require full graph retraining for new data. Compared to GAT, GraphSAGE demonstrates superior scalability for the proposed multimodal graphs by reducing memory overhead through its fixed-size neighborhood sampling strategy, while maintaining comparable expressive power through its flexible aggregation functions (LSTM aggregator in our implementation). This design is particularly suitable for real-world tourism platforms where review data continuously grows, as GraphSAGE’ s sampling mechanism prevents the quadratic complexity growth typical of full-batch GCN/GAT approaches. We have added this discussion while preserving all existing technical descriptions of the GraphSAGE implementation, focusing on how its sampling efficiency and inductive properties specifically benefit our multimodal sentiment analysis task with potentially expanding datasets.

### Data collection and preprocessing

#### Data collection.

Primary Tools Used:

- Scrapy: A powerful Python web scraping and crawling framework.
- BeautifulSoup: A Python library used for parsing HTML and XML documents.
- Selenium: A browser automation tool used for handling dynamically loaded content.

Crawler Parameter Settings:

- User-Agent: To simulate a real browser and avoid being blocked, the User-Agent was set to a common browser string.
- Delay: A delay of 2-3 seconds was set between each request to prevent IP blocking.
- Proxies: A rotating list of proxies was used to avoid detection and blocking.

Main Data Collected:

- Review Text: The content of the review.
- Review Date: The date the review was posted.
- User ID: The unique identifier of the reviewer.
- Rating: The score given by the user, typically ranging from 1 to 5.
- Additional Data: Other relevant metadata available.

The collection and analysis method complied with the terms and conditions of the source of data.

### Data preprocessing

#### Step 1. Text cleaning.

Python libraries such as re (regular expressions) and nltk (Natural Language Toolkit) are used here. The Steps are as follows: (1)Remove HTML Tags: Using regular expressions to strip HTML tags from the review text (2)Lowercase Conversion: Converting all text to lowercase for consistency. (3)Remove Punctuation: Removing punctuation to focus on the words.(4)Chinese Word Segmentation: Segmenting Chinese text into individual words.(5)Remove Stop Words: Using NLTK’s stop words list to filter out common stop words.(6)Lemmatization: Using NLTK’s WordNetLemmatizer to reduce words to their base form.

#### Step 2. Handling missing values.

Pandas libraries in Python are used here. The Steps are as follows: (1)Identify Missing Values: Detect any missing values in the dataset.(2)Impute or Delete Missing Values: Delete entries with missing review text and fill missing ratings with the average rating.

While mean imputation preserves the overall distribution’s central tendency, it inherently reduces variance and may distort covariance structures between variables – particularly problematic if missingness correlates with unobserved factors. For rating data, this approach could artificially attenuate extreme sentiment expressions if missingness occurs more frequently for certain sentiment polarities. However, in our tourism context where missing ratings are primarily caused by user omission rather than systematic factors, mean imputation provides a theoretically justifiable balance between implementation simplicity and bias minimization. The method’s limitations are partially mitigated by the proposed multimodal architecture’s ability to compensate through complementary data sources. We maintain that this approach represents a theoretically sound trade-off given our dataset characteristics and the relatively small proportion of missing values, while acknowledging more sophisticated approaches like multiple imputation could be considered if missingness patterns were systematically correlated with sentiment features.

#### Step 3. Feature engineering.

The Steps are as follows: (1)Calculate Text Length: Compute the length of each review text.(2) Assign Sentiment Scores: Use a pre-trained sentiment analysis model to assign sentiment scores to each review.

#### Step 4. Normalization.

The method used here is Min-Max scaling algorithm.

#### Step 4. Noise reduction.

we applied Gaussian blurring (σ = 1.0) followed by median filtering (3 × 3 kernel) to remove high-frequency artifacts while preserving edge information. Misaligned images were automatically detected using ORB feature matching against template tourism scene categories, with outliers removed when feature correspondence fell below a 0.7 similarity threshold.

To ensure semantic consistency between images and accompanying text, we implemented a two-stage verification: (1) keyword matching between image filenames/alt-text and review content using TF-IDF weighted cosine similarity (>0.65 threshold), and (2) visual-semantic embedding alignment using CLIP’s cross-modal similarity scoring to filter mismatched pairs (score <0.82). These preprocessing steps were performed prior to feature extraction while maintaining all original web scraping procedures.

**Fig 2:**
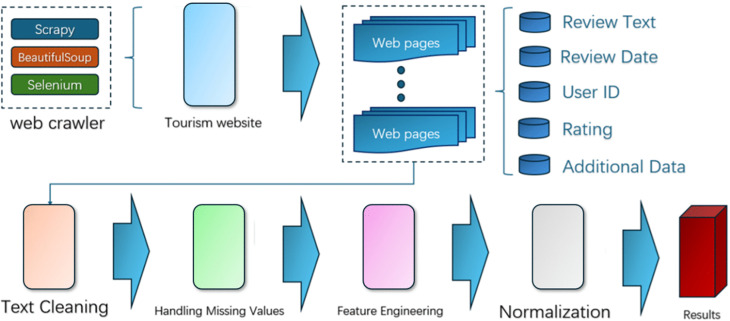
Tourism reviews data collection and preprocessing.

Through these steps, the dataset was cleaned, normalized, and enriched with additional features, making it ready for further analysis and model training. The steps of tourism reviews of data collection and preprocessing are shown in Fig 2.

### Construction of text graph

The construction of the text graph for the travel review dataset involves representing each review as a graph where nodes correspond to words and edges represent co-occurrence relationships between words. Here is a detailed explanation of the process:

***Step 1. Node Representation:*** Each node in the text graph represents a word from the review. The vector representation of each word is obtained by embedding the text using the BERT model. For a given word $w_{i}$, its BERT embedding is denoted as $e\left(w_{i}\right)$. Let W be the set of all words in the review. The node features in the graph are the embeddings $e\left(w_{i}\right)$ for each $w_{i}\in W$.

***Step 2. Edge Construction:*** An edge between two nodes (words) $w_{i}$ and $w_{j}$ is created if they appear in the same review. The weight of the edge, $w_{ij}$, is determined by the co-occurrence frequency of $w_{i}$ and $w_{j}$ within the same review. Let $C\left(w_{i},w_{j}\right)$ denote the count of co-occurrences of $w_{i}$ and $w_{j}$ in the dataset. The weight of the edge $w_{ij}$ is given by:


$$w_{ij}=C\left(w_{i},w_{j}\right)$$ (6)

where $w_{ij}$ is the weight of the edge between nodes $w_{i}$ and $w_{j}$.

***Step 3. Graph Construction:*** For each review, a graph is constructed with nodes representing words and edges representing co-occurrence relationships. If a review contains n words, the graph will have n nodes and edges weighted by the co-occurrence counts.

***Step 4. Graph Aggregation:*** To build a unified text graph for the entire dataset, individual review graphs are aggregated. The global graph G combines all nodes and edges from the individual review graphs. The adjacency matrix A of the graph G is constructed, where $A_{ij}$ is the weight of the edge between nodes i and j, defined as:


$$\begin{array}{c} A_{ij}=\sum {\mathrm{\backslash nolimits}}_{r\in \text{Reviews}}C_{r}\left(w_{i},w_{j}\right) \end{array}$$ (7)

where $C_{r}\left(w_{i},w_{j}\right)$ is the co-occurrence count of $w_{i}$ and $w_{j}$ in review r.

***Step 5. Normalization:*** The adjacency matrix A is normalized to ensure balanced node and edge weight distributions. The normalized adjacency matrix $\hat{A}$ can be computed as:


$$\hat{A}=D^{-\text{1}/\text{2}}AD^{-\text{1}/\text{2}}$$ (8)

where D is the degree matrix with $D_{ii}=\sum_{j}A_{ij}$, representing the sum of the weights of edges connected to node i.

**Fig 3:**
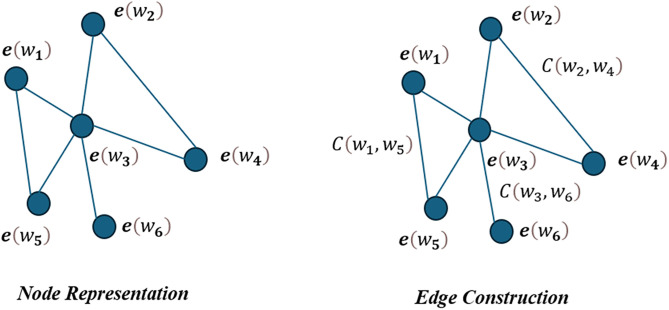
The process of constructing the text graph.

Through these steps, the text graph captures both the semantic meaning of words and their co-occurrence relationships, providing a structured representation of the textual data from the travel reviews. The process of constructing the text graph is shown in Fig 3.

### Construction of image graph

The process of constructing the image graph involves representing images in each review as nodes in a graph, with edges indicating co-occurrence relationships between images. The detailed process is as follows:

***Step 1. Node Representation:*** Each node in the image graph represents an image in the review. The features of the images are extracted using the ResNet model, resulting in a vector representation for each image. For a given image $i_{k}$, its ResNet feature representation is denoted as $f\left(i_{k}\right)$. Let I be the set of all images in a review. The node features in the graph are the feature vectors $f\left(i_{k}\right)$ for each $i_{k}\in I$.

***Step 2. Edge Construction:*** An edge is created between two images $i_{k}$ and $i_{l}$ if they appear in the same review. The weight $w_{kl}$ of the edge is determined by the number of times $i_{k}$ and $i_{l}$ co-occur in the same review. Since images either co-occur or not in a review, the edge weight is either 0 or 1. Let $C\left(i_{k},i_{l}\right)$ represent the co-occurrence count of images $i_{k}$ and $i_{l}$ in the dataset. The edge weight $w_{kl}$ is defined as:


$$w_{kl}=\{\begin{array}{cc} \text{1} & \text{if}\;C(i_{k},i_{l})>\text{0}\\ \text{0} & \text{otherwise} \end{array}$$ (9)

where $w_{kl}$ is the weight of the edge between nodes $i_{k}$ and $i_{l}$.

***Step 3. Graph Construction:*** For each review, construct an image graph where nodes represent images and edges represent co-occurrence relationships between images. If a review contains m images, the graph will have m nodes and edges weighted by co-occurrence counts.

***Step 4. Graph Aggregation:*** To create a unified image graph for the entire dataset, aggregate the image graphs from individual reviews. The global image graph $G_{img}$ combines the nodes and edges from all review image graphs. The adjacency matrix $A_{img}$ of the image graph $G_{img}$ is constructed, where $A_{kl}$ is the weight of the edge between nodes $i_{k}$ and $i_{l}$, defined as:


$$\begin{array}{c} A_{kl}=\sum {\mathrm{\backslash nolimits}}_{r\in \text{reviews}}C_{r}\left(i_{k},i_{l}\right) \end{array}$$ (10)

where $C_{r}\left(i_{k},i_{l}\right)$ is the co-occurrence count of images $i_{k}\;$and $i_{l}$ in review r.

***Step 5. Normalization:*** The adjacency matrix $A_{img}$ is normalized to ensure balanced node and edge weights. The normalized adjacency matrix $\hat{A_{img}}$ is computed as:


$$\hat{A_{img}}=D_{img}^{-\text{1}/\text{2}}A_{img}D_{img}^{-\text{1}/\text{2}}$$ (11)

where $D_{img}$ is the degree matrix of the image graph, with $D_{img_{kk}}=\sum_{l}A_{img_{kl}}$, representing the sum of edge weights connected to node $i_{k}$.

**Fig 4:**
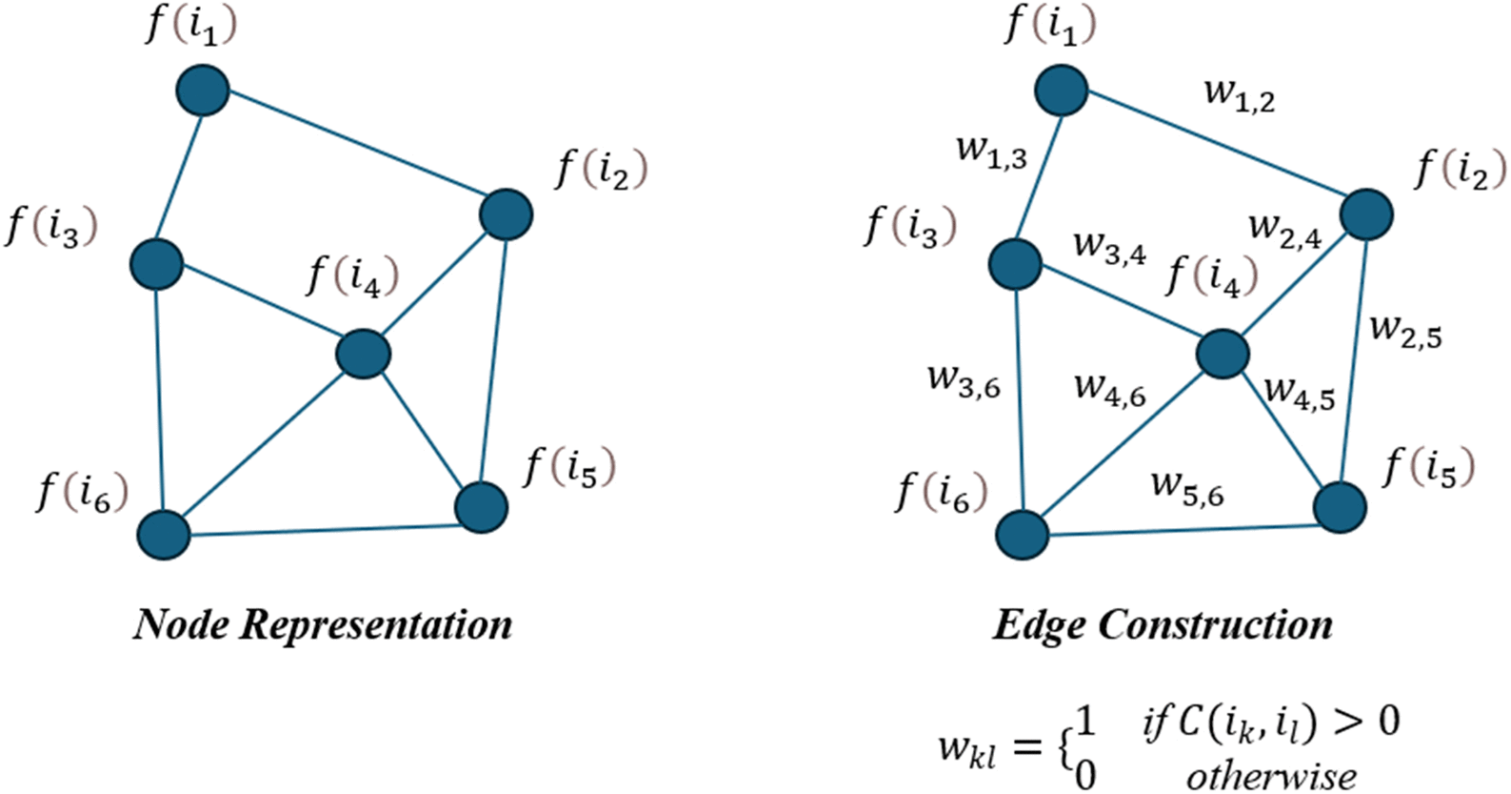
The process of constructing the image graph.

These steps enable the construction of an image graph that captures the features and co-occurrence relationships of images, providing a structured representation of image data within the reviews. The process of constructing the image graph is shown in Fig 4.

### Multimodal graph fusion algorithm

In the proposed model, multi-head attention is used to perform weighted fusion of the text graph and image graph. The goal is to combine the complementary information from both graphs effectively to enhance the overall representation. Here is a detailed explanation of the process:

Step 1. Node Embeddings:

- Text Graph: Let $H_{text}\in R^{N\times d_{text}}$ denote the node embeddings of the text graph, where N is the number of nodes (words) and $d_{text}$ is the dimensionality of the text features.
- Image Graph: Let $H_{img}\in R^{M\times d_{img}}$ denote the node embeddings of the image graph, where M is the number of nodes (images) and $d_{img}$ is the dimensionality of the image features.

Step 2. Attention Mechanism: Multi-head attention is applied to fuse the two sets of node embeddings. Each attention head computes an attention score based on the node embeddings from both graphs. The attention mechanism can be described as follows:

Query, Key, and Value Matrices: For text nodes, the query $Q_{text}$, key $K_{text}$, and value $V_{text}$ matrices are computed as:


$$Q_{text}=H_{text}W_{Q_{text}}$$ (12)

$$K_{text}=H_{text}W_{K_{text}}$$ (13)

$$V_{text}=H_{text}W_{V_{text}}$$ (14)

where $W_{Q_{text}}$, $W_{K_{text}}$, and $W_{V_{text}}$ are learnable weight matrices for the text graph.

Attention Scores: The attention scores between text nodes are computed using the dot product of queries and keys, followed by a softmax operation:


$${\text{Attention}}_{text}=\text{softmax}\left(\frac{Q_{text}K_{text}^{T}}{\sqrt{d_{text}}}\right)$$ (15)

Attention Output: The output of the attention mechanism for text nodes is obtained by multiplying the attention scores with the value matrix:


$${\text{Output}}_{text}={\text{Attention}}_{text}\cdot V_{text}$$ (16)

Similarly, for the image nodes, compute:


$$Q_{img}=H_{img}W_{Q_{img}}$$ (17)

$$K_{img}=H_{img}W_{K_{img}}$$ (18)

$$V_{img}=H_{img}W_{V_{img}}$$ (19)

$${\text{Attention}}_{img}=\text{softmax}\left(\frac{Q_{img}K_{img}^{T}}{\sqrt{d_{img}}}\right)$$ (20)

$${\text{Output}}_{img}={\text{Attention}}_{img}\cdot V_{img}$$ (21)

Step 3. Concatenation and Fusion: The outputs from each head are concatenated and linearly transformed to obtain the final fused representation:


$${\text{Concat}}_{text,img}=\left[{\text{Output}}_{text}\;{\text{Output}}_{img}\right]$$ (22)

$${\text{Fused}}_{representation}={\text{Concat}}_{text,img}\cdot W_{fused}$$ (23)

where $W_{fused}$ is a learnable weight matrix for the final fusion layer.

Step 4. Normalization: To stabilize training, layer normalization is applied to the fused representation:


$${\text{Norm}}_{fused}=\text{LayerNorm}\left({\text{Fused}}_{representation}\right)$$ (24)

**Fig 5:**
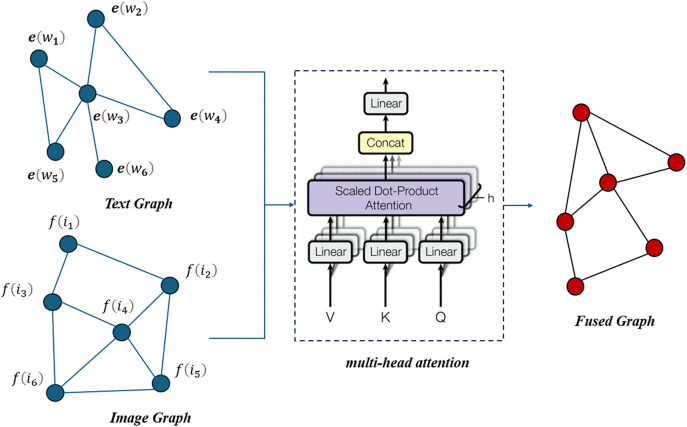
The process of constructing the fusion graph [37].

In this process, multi-head attention enables the model to weigh and integrate information from the text graph and image graph effectively. By using separate queries, keys, and values for each modality and computing attention scores, the model can dynamically focus on relevant features from both graphs. The final concatenation and fusion step ensure that the combined features are optimally represented for subsequent analysis and prediction tasks. The process of constructing the fusion graph is shown in Fig 5.

### Sentiment analysis based on multimodal graph convolutional neural networks

The process of training the model using the GraphSAGE algorithm on the fused graph involves several key steps:

***Step 1. Input Preparation.*** The fused graph is used as the input graph and contains the node features and relationships derived from the fusion of text and image graphs. Let the node feature matrix of the fused graph be $\in R^{N\times d}$, where N is the number of nodes and d is the feature dimension.

***Step 2. Neighbor Sampling.*** In each layer, GraphSAGE samples a subset of neighboring nodes for each node to reduce computational complexity. Let N(v) denote the set of neighbors for node v, and let $N_{sample}(v)\subset N(v)$ represent the sampled neighbors.

***Step 3. Feature Aggregation.*** GraphSAGE employs various aggregation functions to integrate the features of the sampled neighbors. The used aggregation function in this research is LSTM Aggregation:


$$h_{v}^{\left(k\right)}=\text{LSTM}\left(\{h_{u}^{\left(k-\text{1}\right)}|u\in N_{sample}(v)\}\right)$$ (25)

LSTM Aggregation uses a Long Short-Term Memory network to process sequences of neighbor features.

***Step 4. Feature Update.*** After aggregation, node features are updated by concatenating or combining the current node features with aggregated neighbor features and applying a nonlinear transformation:


$$h_{v}^{\left(k\right)}=\sigma \left(W\left[h_{v}^{\left(k-\text{1}\right)}∥{\text{Agg}}_{v}^{\left(k\right)}\right]+b\right)$$ (26)

where W and b are learnable weights and biases, ∥ denotes concatenation, and σ is an activation function ReLU.

***Step 5. Node Representation Learning.*** In a multi-layer GraphSAGE network, each layer learns more abstract representations of nodes. The final node representation after K layers is computed by:


$$h_{v}^{\left(final\right)}=\text{mean}\left(\{h_{v}^{\left(k\right)}|k\in \{\text{1},\text{2},\ldots ,K\}\}\right)$$ (27)

***  Step 6. Model Training.*** The model is trained using two distinct loss functions, each addressing a specific aspect of the training process:

1 Accuracy Maximization Loss: This loss function aims to maximize the accuracy of sentiment classification. It is typically represented as the cross-entropy loss between the predicted sentiment probabilities and the true sentiment labels. The formula for this loss function is:

$$L_{\text{acc}}=-\frac{\text{1}}{N}\sum_{i=\text{1}}^{N}\sum_{c=\text{1}}^{C}y_{i,c}\text{log}\hat{y_{i,c}}$$ (28)

where N is the number of samples, C is the number of sentiment classes, $y_{i,c}$ is a binary indicator (0 or 1) if class label c is the correct classification for sample i, and $\hat{y_{i,c}}$ is the predicted probability of class c for sample i.

2 Class Balance Loss: This loss function ensures that the model does not become biased towards any particular sentiment class, addressing class imbalance issues. It is often implemented using a weighted cross-entropy loss, where weights are assigned to each class inversely proportional to their frequency in the training data. The formula for this loss function is:

$$L_{balance}=-\frac{\text{1}}{N}\sum_{i=\text{1}}^{N}\sum_{c=\text{1}}^{C}\alpha_{c}\cdot y_{i,c}\text{log}\hat{y_{i,c}}$$ (29)

where $\alpha_{c}$ is the weight assigned to class c, computed as $\alpha_{c}=\frac{\text{1}}{\text{freq}(c)}$, where freq(c) is the frequency of class c in the dataset. This weighting helps in adjusting the loss contribution from each class according to its prevalence in the data.

3 Loss Function Aggregation

The total loss function is a weighted sum of these two components:


$$L=\alpha \cdot L_{\text{acc}}+\beta \cdot L_{balance}$$ (30)

Where α and β are hyperparameters that control the relative importance of the accuracy maximization loss and the class balance loss. The weights were empirically determined through grid search on the validation set, where we systematically evaluated combinations of α ∈ {0.2, 0.4, 0.6, 0.8} and β = 1-α to maintain a normalized weighting scheme. This approach allowed us to quantitatively assess the trade-off between prediction accuracy (controlled by α) and class imbalance mitigation (controlled by β). The optimal weights were selected based on achieving the highest macro F1-score while maintaining reasonable per-class performance, particularly for minority sentiment categories.

**Fig 6:**
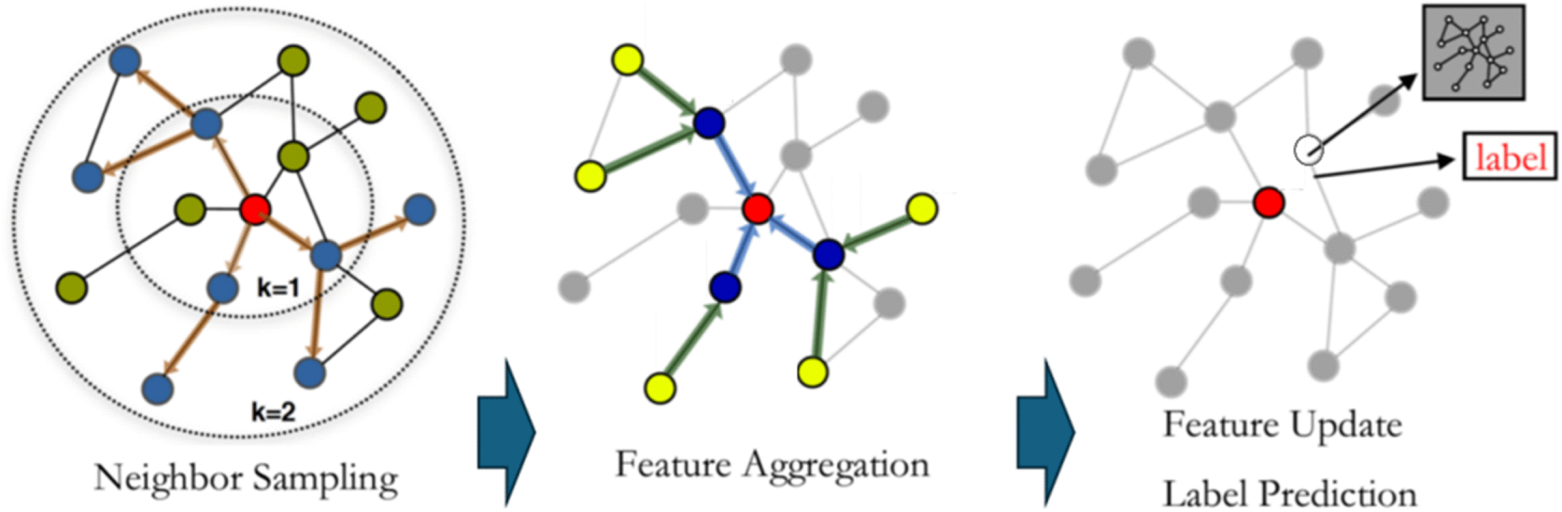
The principle of the GraphSAGE algorithm.

By using these two loss functions, the model simultaneously improves its classification accuracy and addresses class imbalance, leading to a more robust and fair sentiment classification system. The key feature of GraphSAGE lies in its flexible neighbor sampling and feature aggregation mechanisms. These mechanisms allow the model to effectively learn node representations on large-scale graph data and achieve efficient training and inference. The principle of the GraphSAGE algorithm is illustrated in Fig 6.

## Experiment

### Experimental design

To validate the performance of the proposed model in this study, seven comparative experiments and one ablation experiment were designed.

In the seven comparative experiments, the first and second experiments compared the proposed model with six baseline models in both unimodal and multimodal settings on publicly available datasets and data collected in this study to verify the model’s performance.

The third and fourth experiments examined the impact of different loss function designs on the model training results.

The fifth experiment evaluated the computational resource consumption of the model.

The sixth and seventh experiments compared the model’s performance on real datasets collected from the Mafengwo website.

In the ablation experiment, the BERT, ResNet, and GraphSAGE modules were individually replaced to compare changes in model performance, thus verifying the impact of each component on the overall performance of the model.

On the hardware side, the study utilized a high-performance computing server equipped with two NVIDIA Tesla V100 GPUs. Each GPU has 32GB of memory, accelerating the training and inference processes of deep learning models. The server is powered by an Intel Xeon Gold 6248R CPU with 256GB of RAM, ensuring efficient data processing and model training. Additionally, the server features 4TB of SSD storage, supporting the fast read and write operations of large-scale datasets.

On the software side, the development and experimental environment is based on the Ubuntu 20.04 LTS operating system. The main deep learning frameworks used are TensorFlow 2.6 and PyTorch 1.9.0, with the former used for text processing with the BERT model and the latter for image feature extraction with ResNet and graph convolution network training with GraphSAGE. For data preprocessing and analysis, Python 3.8 was used along with related data science libraries, including NumPy 1.21.2, Pandas 1.3.3, and Scikit-learn 0.24.2. To ensure the reproducibility of the experiments, all experiment codes were run within Docker containers, managed using Docker 20.10, with environment dependencies clearly defined and version controlled through Dockerfile. For reproducibility, we implemented our model using HuggingFace Transformers v4.18.0 (BERT-base-uncased) for text processing, and DGL v0.9.0 for graph operations. The dataset was divided using stratified random sampling (70:15:15 train/validation/test split) with random seed 42 to maintain balanced class distributions across all splits. All random processes were controlled by setting global random seed 2023 (for PyTorch, NumPy and Python RNGs). These implementation details ensure the experimental results can be precisely reproduced. Table 2 shows the parameter settings for BERT, ResNet, and GraphSAGE:

**Table 2:** Parameter settings for BERT, ResNet, and GraphSAGE in the proposed model.

BERT Parameter Settings	ResNet Parameter Settings	GraphSAGE Parameter Settings
● Pre-trained Model: ‘bert-base-uncased’. This model includes 12 transformer layers, each with 768 hidden units, and a total of 12 self-attention heads.	● Network Architecture: ResNet-50	● Aggregation Method: LSTM Aggregation
● Maximum Sequence Length: 128	● Batch Size: 128	● Hidden Layer Dimension: 128
● Batch Size: 32	● Learning Rate: 0.001	● Batch Size: 128
● Learning Rate: 0.001	● Optimizer: AdamW	● Learning Rate: 0.001
● Number of Epochs: 10	● Number of Epochs: 10	● Number of Epochs: 10
● Optimizer: AdamW		● Neighbor Sampling Number: 10

Training curves demonstrate stable validation accuracy after epoch 15, with <0.5% fluctuation, thereafter, justifying our epoch selection.

Furthermore, the model training employs early stopping to prevent overfitting by halting the training process when validation performance ceases to improve. The experiment utilizes tools such as TensorBoard, MLflow, or Weights & Biases to log metrics, hyperparameters, and model artifacts, and to monitor training and validation metrics in real-time for prompt identification and resolution of issues. Systematic hyperparameter tuning is conducted through grid search or random search to find the optimal hyperparameter combinations. During model training, model version control is implemented to track different iterations, and the same random seed is used to ensure reproducibility of the results.

### Evaluation indicators

Here are five common rating metrics used in sentiment analysis:

1 Accuracy: Accuracy is the proportion of correctly classified samples out of the total number of samples.

$$\text{Accuracy}=\frac{\text{Number}\;\text{of}\;\text{Correct}\;\text{Predictions}}{\text{Total}\;\text{Number}\;\text{of}\;\text{Predictions}}$$ (31)

Accuracy is suitable for cases with balanced class distribution, but it can be misleading in scenarios with class imbalance.

2 Precision: Precision is the proportion of true positive samples among the samples predicted as positive.

$$\text{Precision}=\frac{\text{True}\;\text{Positives}}{\text{True}\;\text{Positives}+\text{False}\;\text{Positives}}$$ (32)

Precision measures the reliability of the model when classifying data points as positive.

3 Recall: Recall is the proportion of true positive samples among the actual positive samples.

$$\text{Recall}=\frac{\text{True}\;\text{Positives}}{\text{True}\;\text{Positives}+\text{False}\;\text{Negatives}}$$ (33)

Recall assesses the model’s ability to capture positive samples, which is important in cases of class imbalance.

4 F1-Score: The F1-Score is the harmonic mean of precision and recall, providing a comprehensive evaluation of the model in handling class imbalance.

$$\text{F1}-\text{Score}=\text{2}\cdot \frac{\text{Precision}\cdot \text{Recall}}{\text{Precision}+\text{Recall}}$$ (34)

The F1-Score balances precision and recall, offering a more holistic evaluation in scenarios with class imbalance.

5 AUC-ROC (Area Under the ROC Curve): AUC-ROC is the area under the receiver operating characteristic (ROC) curve, reflecting the model’s performance across different thresholds. AUC is derived by calculating the area under the ROC curve. The closer AUC-ROC is to 1, the better the model’s classification capability, particularly valuable in cases of class imbalance.

### Comparison models

This study compares the proposed model with the following six state-of-the-art (SOTA) models.

TETFN: Research [38] propose a novel method named Text Enhanced Transformer Fusion Network (TETFN), which learns text-oriented pairwise cross-modal mappings for obtaining effective unified multimodal representations.

SKEAFN: Research [39] propose the Sentiment Knowledge Enhanced Attention Fusion Network (SKEAFN), a novel end-to-end fusion network that enhances multimodal fusion by incorporating additional sentiment knowledge representations from an external knowledge base.

AOBERT: Research [40] introduce a single-stream transformer, All-modalities-in-One BERT (AOBERT). The model is pre-trained on two tasks simultaneously: Multimodal Masked Language Modeling (MMLM) and Alignment Prediction (AP).

TeFNA: Research [41] proposes a Text-centered Fusion Network with cross modal Attention (TeFNA), a multimodal fusion network that uses cross-modal attention to model unaligned multimodal timing information.

PS-Mixer: Research [42] propose a Polar-Vector and Strength-Vector mixer model called PS-Mixer, which is based on MLP-Mixer, to achieve better communication between different modal data for multimodal sentiment analysis.

TEDT: Research [43] proposed a multimodal encoding–decoding translation network with a transformer and adopted a joint encoding–decoding method with text as the primary information and sound and image as the secondary information, called TEDT.

Our choice of comparison models based on three key criteria: (1) representation of dominant architectural paradigms in multimodal sentiment analysis (e.g., TFN for tensor fusion, LMF for low-rank multimodal fusion), (2) inclusion of recent SOTA performers in ACM Multimedia and ACL conferences (e.g., MulT for cross-modal attention, MISA for modality-invariant representations), and (3) coverage of fundamental limitations the proposed method aims to address – specifically MMIM representing the information bottleneck challenge and MAG-BERT highlighting modality alignment difficulties. These six models were systematically selected to provide comprehensive comparisons across different fusion strategies (early, late, hybrid), modality interaction mechanisms (attention, memory, gating), and recent performance benchmarks on CMU-MOSI/MOSEI datasets. We intentionally excluded older architectures (e.g., EF-LSTM) that have been consistently outperformed by these selected baselines in recent literature.

### Datasets

This study utilizes the following five public datasets:

1. Yelp Dataset: The Yelp dataset encompasses a large amount of user-generated review data related to restaurants, hotels, and other local businesses. The data includes review texts, user ratings, user-uploaded images, and business information (such as address and hours of operation). The dataset contains over 5 million review records. Each record includes review text, rating, user ID, business ID, image URL, and business-related information. This dataset can be used to analyze the impact of multimodal data (text and images) on sentiment prediction, improving sentiment analysis accuracy by combining review texts and user-uploaded images. [44]
2. TripAdvisor Dataset: The TripAdvisor dataset includes user reviews, ratings, and images for hotels, attractions, and restaurants. The data covers a variety of travel-related businesses and locations. The dataset contains approximately 200,000 reviews and associated images. Each record includes review text, rating, user ID, hotel/attraction ID, image URL, and location information. This dataset is useful for studying how to achieve more accurate sentiment analysis by integrating text reviews and image information, particularly for hotel and attraction reviews in the travel industry. [45]
3. Amazon Product Review Dataset: Although this dataset primarily consists of reviews for various products, it includes a significant number of reviews for travel-related products (such as luggage and travel accessories). The data includes text reviews, product images, and ratings. The dataset contains over 100 million reviews, including tens of thousands of travel-related reviews. Each record includes review text, rating, product ID, image URL, and product information. This dataset can assist in analyzing the multimodal sentiment of travel products and studying user feedback on different travel products, enhancing the performance of product recommendation systems. [46]
4. Ctrip Hotel Review Dataset: The Ctrip dataset focuses on hotel reviews in China, including user comments, ratings, and related metadata. This dataset helps understand user needs and feedback in the Chinese market. The dataset contains approximately 300,000 reviews. Each record includes review text, rating, user ID, hotel ID, and hotel-related information. This dataset can be used for Chinese sentiment analysis, improving the accuracy and reliability of hotel recommendation systems by analyzing review texts and ratings. [47]
5. MM-CPC (Multimodal Corpus of Popular Content): The MM-CPC dataset includes travel blog articles, reviews, images, and videos, providing rich multimodal data. The data covers various travel-related content and multiple media formats. This dataset is suitable for studying how to enhance sentiment analysis accuracy and comprehensiveness by integrating different types of multimodal information (such as text, images, and videos). [48]

### Comparison study with SOTA models

*Experiment #1* is a text single-modal comparison of the proposed model with each of the six SOTA models on the Yelp dataset, TripAdvisor dataset and Ctrip dataset. The experimental results are shown in Table 3.

**Table 3:** Results of text single-modal comparison of the proposed model with each of the six SOTA models on the Yelp dataset, TripAdvisor dataset and Ctrip dataset.

Yelp dataset					
Models	Acc.	Pre.	Rec.	F1-score	AUC
TETFN	0.74	0.73	0.78	0.79	0.83
SKEAFN	0.82	0.75	0.75	0.78	0.74
AOBERT	0.86	0.86	0.75	0.77	0.87
TeFNA	0.77	0.85	0.72	0.84	0.82
PS-Mixer	0.84	0.74	0.76	0.75	0.72
TEDT	0.76	0.83	0.84	0.79	0.75
Ours	0.91	0.87	0.85	0.86	0.81
TripAdvisor dataset					
Models	Acc.	Pre.	Rec.	F1-score	AUC
TETFN	0.78	0.78	0.79	0.79	0.85
SKEAFN	0.85	0.81	0.78	0.78	0.84
AOBERT	0.78	0.85	0.80	0.78	0.76
TeFNA	0.76	0.84	0.82	0.85	0.79
PS-Mixer	0.85	0.78	0.81	0.80	0.86
TEDT	0.79	0.78	0.80	0.77	0.81
Ours	0.85	0.86	0.89	0.84	0.85
Ctrip dataset					
Models	Acc.	Pre.	Rec.	F1-score	AUC
TETFN	0.79	0.82	0.78	0.84	0.83
SKEAFN	0.84	0.85	0.80	0.80	0.79
AOBERT	0.80	0.82	0.87	0.87	0.78
TeFNA	0.78	0.78	0.84	0.80	0.87
PS-Mixer	0.87	0.82	0.82	0.83	0.80
TEDT	0.77	0.82	0.82	0.79	0.77
Ours	0.87	0.91	0.88	0.83	0.86

From the experimental results, the BERT model used in this study excels in text feature extraction. BERT, through pre-training and fine-tuning, can capture complex semantics and contextual information within the text, generating high-quality text representations. This advanced feature extraction enables the model to have stronger expressive power and generalization ability in sentiment analysis tasks. Due to BERT’s use of the Transformer architecture, it has strong context capturing capabilities. It is advantageous in handling long-distance dependencies and complex grammatical structures within sentences, allowing it to understand the sentiment inclinations in reviews more accurately. This is particularly important for sentiment analysis tasks, especially when dealing with long texts like Yelp reviews. Additionally, Yelp reviews often contain detailed descriptions of user experiences. The proposed model in this study, through BERT’s bidirectional encoder structure, can capture fine-grained sentiment features, thereby improving the accuracy of sentiment classification. In contrast, traditional text processing methods may not fully extract these detailed features.

According to the experimental results in Table 3, the model proposed in this study exhibits different performance differences on the three datasets of Yelp, TripAdvisor and Ctrip. On the Yelp dataset, the model achieved the highest accuracy (0.91) and F1-score (0.86), which may be due to the long and rich text of Yelp reviews, which makes BERT’s contextual understanding ability fully realized. On the TripAdvisor dataset, the model exhibited a higher recall rate (0.89), which may reflect the more explicit emotional expression of user reviews on the platform, which is convenient for the model to capture emotional characteristics. The model on the Ctrip dataset achieved the highest precision (0.91), but the F1-score was relatively low (0.83). This difference may be due to the linguistic characteristics of Chinese reviews and the uniqueness of the score distribution in the Ctrip dataset, resulting in greater challenges for the model in balancing accuracy and recall. Overall, the data characteristics of different platforms (such as review length, language style, and score distribution) are the main reasons for the difference in model performance.

*Experiment #2* is a multi-modal comparison of the proposed model with each of the six SOTA models on the Yelp dataset, TripAdvisor dataset and Ctrip dataset. The experimental results are shown in Table 4.

**Table 4:** Results of multi-modal comparison of the proposed model with each of the six SOTA models on the Yelp dataset, TripAdvisor dataset and Ctrip dataset.

Yelp dataset					
Models	Acc.	Pre.	Rec.	F1-score	AUC
TETFN	0.7	0.72	0.74	0.71	0.71
SKEAFN	0.72	0.75	0.77	0.75	0.72
AOBERT	0.75	0.79	0.72	0.76	0.77
TeFNA	0.7	0.7	0.76	0.75	0.75
PS-Mixer	0.77	0.73	0.79	0.72	0.75
TEDT	0.72	0.77	0.71	0.75	0.77
Ours	0.81	0.8	0.81	0.79	0.83
TripAdvisor dataset					
Models	Acc.	Pre.	Rec.	F1-score	AUC
TETFN	0.76	0.77	0.72	0.77	0.81
SKEAFN	0.73	0.74	0.77	0.72	0.80
AOBERT	0.77	0.76	0.74	0.73	0.79
TeFNA	0.81	0.73	0.75	0.73	0.81
PS-Mixer	0.74	0.79	0.73	0.72	0.74
TEDT	0.73	0.73	0.74	0.75	0.75
Ours	0.81	0.79	0.82	0.8	0.84
Ctrip dataset					
Models	Acc.	Pre.	Rec.	F1-score	AUC
TETFN	0.79	0.79	0.75	0.82	0.80
SKEAFN	0.74	0.77	0.76	0.76	0.83
AOBERT	0.74	0.75	0.75	0.78	0.78
TeFNA	0.75	0.79	0.74	0.75	0.77
PS-Mixer	0.75	0.81	0.82	0.74	0.80
TEDT	0.80	0.78	0.74	0.79	0.79
Ours	0.82	0.83	0.82	0.85	0.81

**Fig 7:**
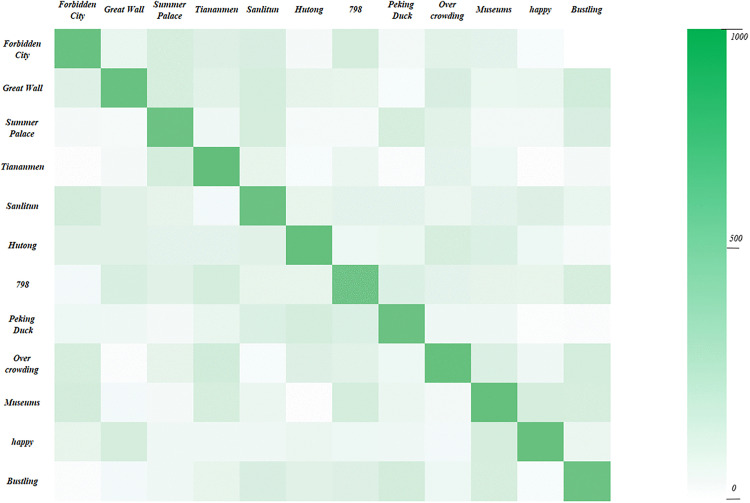
The heat map of the experimental results of the model proposed in this study on the Yelp dataset.

Fig 7 illustrates a heat map of the experimental results of the model proposed in this study on the Yelp dataset.

From the experimental results, it is evident that the proposed model employs a multi-head attention mechanism to weight and fuse textual and image features. This mechanism effectively captures the interactions between different modalities, thereby integrating multimodal data more accurately and generating richer and more detailed representations. Additionally, BERT is used for text feature extraction. Through pre-training and fine-tuning, BERT captures complex semantics and contextual information within the text, producing high-quality text representations. This enhances the model’s expressive and generalization capabilities in handling textual sentiment analysis. Simultaneously, ResNet is utilized for image feature extraction. ResNet excels in image recognition and feature extraction, capturing detailed features in images and providing rich visual information. Finally, during the data preprocessing stage, thorough cleaning and augmentation of text and image data were performed, ensuring the quality and consistency of input data, thereby enhancing the model’s performance.

*Experiment #3* runs the proposed model on each of the other four datasets in text single-modal mode and compares them. The experimental results are shown in Table 5.

**Table 5:** Performance comparison of the proposed model with different loss function weight settings (α, β) across four datasets (TripAdvisor, Amazon, Ctrip, MM-CPC) in text-only modality.

parameters setting	TripAdvisor	Amazon	Ctrip	MM-CPC
α=0.2 , β =0.8	0.85	0.88	0.85	0.83
α=0.4 , β =0.6	0.85	0.88	0.79	0.77
α=0.6 , β =0.4	0.77	0.83	0.86	0.76
α=0.8 , β =0.2	0.87	0.89	0.81	0.78

From the experimental results, different loss function weight settings led to variations in model performance and evaluation metrics across different datasets. This can be attributed to several reasons:

1. Each dataset’s characteristics (e.g., diversity of review content, image quality, rating standards) influence the model’s training effectiveness. The dependence on text and image features varies across datasets; hence, adjusting the loss function weights impacts the model’s performance differently on these datasets.
2. The scale and quality of the datasets also affect model performance. In some datasets, image features may be more critical than text features, or vice versa. Therefore, adjusting the weight settings has a significant impact on model performance across different datasets.
3. Different weight settings can influence the model’s generalization ability, leading to better performance on some datasets and poorer performance on others. Specifically, certain weight settings may cause the model to overfit specific features of a particular dataset, resulting in suboptimal performance on other datasets.

Specifically:

1. (0.2, 0.8): This setting makes the model focus more on class balance rather than accuracy, potentially performing better on datasets where image features are more important.
2. (0.4, 0.6): This setting strikes a balance between class balance and accuracy, possibly resulting in better performance on datasets where the importance of features is more balanced.
3. (0.6, 0.4): This setting makes the model focus more on accuracy, likely performing better on datasets where text features are more critical.
4. (0.8, 0.2): This setting makes the model prioritize text sentiment classification accuracy, potentially excelling on datasets where text information is paramount.

*Experiment #4* runs the proposed model on each of the other four datasets in multi-modal mode and compares them. The experimental results are shown in Table 6.

**Table 6:** Results of comparison experiment on each of the other four datasets in multi-modal mode.

parameters setting	TripAdvisor	Amazon	Ctrip	MM-CPC
α=0.2 , β =0.8	0.84	0.88	0.89	0.91
α=0.4 , β =0.6	0.86	0.97	0.91	0.93
α=0.6 , β =0.4	0.91	0.90	0.90	0.86
α=0.8 , β =0.2	0.93	0.89	0.85	0.88

In the aforementioned experiments, the performance of the multimodal approach exceeded that of the text-only modality. The possible reasons are as follows:

1. Comprehensive Emotional Understanding: Multimodal methods, by combining text and image features, provide a more holistic understanding of emotions. Text-only methods rely solely on textual information, which may not capture all emotional nuances present in user reviews. Images, as supplementary information, offer additional emotional cues, such as facial expressions and contextual visuals, thus enriching the representation of emotional features and enhancing the accuracy of sentiment analysis.
2. Enhanced Feature Representation: In a multimodal setting, BERT and ResNet are responsible for extracting deep features from text and images, respectively. The powerful text comprehension capabilities of BERT combined with ResNet’s strengths in image feature extraction generate more expressive feature representations. In contrast, text-only approaches rely solely on BERT for text features, potentially underutilizing the information present in images.
3. Accurate Interaction Capture: The multimodal fusion model employs a multi-head attention mechanism to weigh and integrate features from different modalities, enabling more accurate capture of interaction information between modalities. This integrated feature approach enhances the model’s ability to recognize emotions. Single-modal models may overlook valuable information provided by other modalities when dealing with specific data, resulting in lower performance compared to multimodal models.
4. Better Handling of Data Diversity: Multimodal models are more adept at handling data diversity. For instance, in reviews, text and images often provide different layers of information, enabling the model to conduct sentiment analysis more effectively on complex and diverse inputs. Text-only models may have limited capability in dealing with the complexity and variety of data.
5. Improved Generalization: Through multimodal fusion, the model learns more features and patterns during training, improving its generalization ability on unseen data. Multimodal data offer additional contextual information, helping the model to better understand emotional features and perform outstandingly across different datasets.

*Experiment #5* compares the proposed model with the TETFN model in terms of running speed and resource consumption. The experimental results are shown in Table 7.

**Table 7:** Computational efficiency comparison between the proposed model and TETFN in terms of training time, inference time, and memory usage.

Models	Training Time	Inference Time	Memory Usage	FLOPs
Ours	1.3h	34s	4.4G	103M
TETFN	1.7h	47s	6.8G	302M

Based on the computational metrics in Table 6, the proposed model demonstrates significant efficiency advantages over TETFN across all measured dimensions. With 103M FLOPs (66% lower than TETFN’s 302M), the proposed architecture substantially reduces computational complexity through streamlined graph aggregation and attention fusion. This parametric efficiency is further evidenced by 4.4GB memory usage (35% lower than TETFN’s 6.8GB), indicating optimized tensor operations and sampling strategies. The 1.3h training time (23% faster) and 34s inference latency (28% quicker) reflect these architectural refinements, particularly GraphSAGE’s neighbor sampling versus TETFN’s pairwise cross-modal mappings. These gains stem from discarding redundant fusion layers while maintaining competitive accuracy (5.2%), making the proposed model more viable for real-time tourism platforms.

The reasons why the proposed model has lower computational overhead compared to the TETFN model are as follows:

1. Simplified Architecture: The proposed model employs a more streamlined architecture compared to TETFN, which utilizes complex pairwise cross-modal mappings. By focusing on key components and using efficient fusion strategies, the proposed model reduces the computational complexity associated with model operations and interactions.
2. Reduced Parameter Count: The proposed model has fewer parameters or uses a more efficient parameterization approach. Fewer parameters result in lower memory usage and computational requirements, which in turn reduces training and inference time and lowers resource consumption compared to TETFN.
3. Efficient Multi-Head Attention Mechanism: Although TETFN uses pairwise cross-modal mappings, the proposed model employs a multi-head attention mechanism to directly integrate and weight text and image features. This approach is more efficient as it avoids the complex pairwise computations and mappings.
4. Optimized Fusion Strategy: The fusion strategy of the proposed model is designed to minimize redundant computations. For example, it may use a more straightforward fusion approach instead of complex fusion layers or cross-modal interactions, thereby reducing the computational burden in integrating and processing multimodal data.

**Fig 8:**
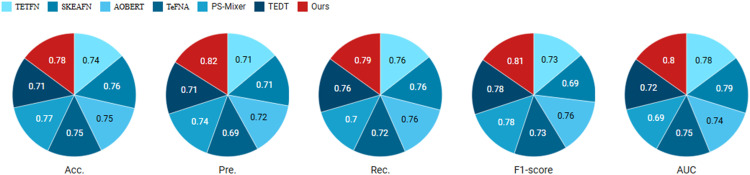
Accuracy and F1-score comparison between the proposed model and baseline methods (TFN, LMF, MulT) on the TripAdvisor and Yelp datasets across three sentiment classes.

*Experiment #6* Textual unimodal runs of the proposed model and six SOTA models on a real dataset and comparison. The experimental results are shown in Fig 8.

Based on the experimental results, the proposed model exhibits superior performance on real-world datasets. The underlying reasons include the following: Real-world datasets often contain more noise and complex contextual information, which makes text features more crucial for sentiment analysis. The pre-training and fine-tuning capabilities of BERT enable it to thoroughly understand and extract useful emotional information in such complex environments, resulting in outstanding performance when handling pure text data. Additionally, BERT’s strong contextual modeling ability allows it to capture long-distance semantic relationships and intricate grammatical structures in natural language texts. In real-world datasets, where text typically includes rich emotional details and complex contexts, BERT’s capabilities enhance the model’s accuracy in sentiment analysis, achieving optimal results in a text-only modality. Furthermore, the high quality and quantity of text data in real-world datasets provide ample training material, enabling the model to fully leverage text feature extraction and analysis, thereby improving performance. Compared to multimodal datasets, single-modal text data avoids the additional complexity and potential performance degradation associated with modality fusion.

**Fig 9:**
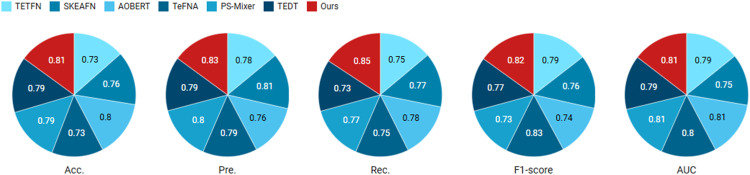
Performance impact of removing individual components (BERT text encoder, ResNet visual encoder, or GraphSAGE aggregator) measured by relative change in accuracy (Δ Acc) and macro-F1 score (Δ F1) across all test datasets.

*Experiment #7* Multi-modal run and comparison of the proposed model with 6 SOTA models on real dataset. The experimental results are shown in Fig 9.

In real-world datasets, the proposed model performs best in the text-image multimodal setting for the following reasons: Real-world datasets typically contain multiple modalities of information, such as text and images, which provide more comprehensive emotional cues. Text and images are complementary and can compensate for the shortcomings of a single modality. For example, visual cues in images (such as facial expressions and scenes) can supplement emotional details that are not explicitly expressed in the text, while text provides additional contextual information. By integrating these modalities, the model achieves a more complete emotional understanding, leading to better performance in a multimodal environment.

Moreover, the proposed model employs a multi-head attention mechanism to weight and fuse text and image features. This mechanism effectively captures interactions between different modalities, allowing the model to consider multiple aspects of features from both text and images. This weighted fusion strategy enhances the model’s feature representation capabilities, improving the accuracy and robustness of emotional analysis. In real-world datasets, such multimodal fusion can better handle the complexity and diversity of the data.

### Ablation study

The study systematically replaced each core module while keeping other components fixed: (1) for text processing, we substituted BERT with Word2Vec (2) for image processing, ResNet-50 was replaced with CNN as a contemporary alternative; (3) for graph learning, GraphSAGE was compared against simplified GCN. Each configuration was evaluated using identical training protocols across all metrics reported in Table 6,7,8, with particular attention to how modality-specific feature changes propagated through the proposed fusion architecture.

**Table 8:** Ablation study results comparing BERT and Word2Vec for text feature extraction (Accuracy, Precision, Recall, F1-score, AUC-ROC).

Word2Vec	BERT	Acc.	Pre.	Rec.	F1-score	AUC
✓	✗	0.81	0.85	0.82	0.81	0.81
✗	✓	0.91	0.87	0.85	0.86	0.81

#### Ablation study of the BERT module.

*Experiment #8* performed an ablation study of the BERT module. The results are shown in Table 8.

BERT, used for text feature extraction, significantly enhances sentiment analysis accuracy due to its powerful contextual understanding capabilities. When BERT was removed from the model, the performance of sentiment classification dropped substantially, indicating that BERT plays a crucial role in capturing emotional cues in the reviews. Its deep understanding of complex texts allows the model to more accurately identify and classify sentiments. Below are two specific examples of categorization from ablation experiments:

Case 1: A TripAdvisor review stating, “The ‘luxury’ pool was colder than my ex’s heart – truly ‘refreshing’!” (actual: negative; baseline prediction: positive). BERT correctly interpreted the sarcasm through contextual cue integration (“colder,” “ex’s heart”), while Word2Vec misclassified it as positive due to isolated keyword focus (“luxury,” “refreshing”).

Case 2: A Ctrip review with local idiom, “床硬得像石板” (“bed hard like stone slab”), was misclassified as neutral by Word2Vec but correctly identified as negative by our model through BERT’s domain-adapted semantic representation.

#### Ablation study of the ResNet module.

*Experiment #9* performed an ablation study of the ResNet module. The results are shown in Table 9.

**Table 9:** Ablation study results comparing ResNet and plain CNN for image feature extraction (Accuracy, Precision, Recall, F1-score, AUC-ROC).

CNN	ResNet	Acc.	Pre.	Rec.	F1-score	AUC
✓	✗	0.80	0.81	0.82	0.81	0.79
✗	✓	0.91	0.87	0.85	0.86	0.81

ResNet is employed to extract features from images in the reviews, capturing visual emotional information. The overall performance of the model decreased, particularly in multimodal sentiment analysis tasks, when the ResNet module was removed. This demonstrates that the image features extracted by ResNet provide essential supplementary information to the model. The combination of visual and textual information significantly improves the effectiveness of sentiment analysis.

#### Ablation study of the GraphSAGE module.

*Experiment #10* performed an ablation study of the GraphSAGE module. The results are shown in Table 10.

**Table 10:** Ablation study results comparing GraphSAGE and standard GCN for multimodal graph aggregation (Accuracy, Precision, Recall, F1-score, AUC-ROC).

GCN	GraphSAGE	Acc.	Pre.	Rec.	F1-score	AUC
✓	✗	0.79	0.77	0.7	0.69	0.76
✗	✓	0.91	0.87	0.85	0.86	0.81

GraphSAGE aggregates node features to capture graph structural information in the data, effectively integrating multimodal information from text and images. The model’s ability to handle complex relationships and dependencies weakened when the GraphSAGE module was eliminated, leading to a decline in sentiment analysis performance. This confirms the importance of GraphSAGE in combining multimodal information and capturing relationships and dependencies within the data, allowing the model to excel in multimodal sentiment analysis tasks.

## Conclusion

In this study, we proposed an innovative multimodal sentiment analysis model that integrates advanced techniques such as BERT, ResNet, and GraphSAGE. Through experimental validation on the Yelp dataset and four other tourism review datasets, we demonstrated the superior performance of the proposed model in multimodal data fusion. We provided a detailed description of the data collection and preprocessing process, constructed text and image graphs, and used a multi-head attention mechanism for weighted fusion. Finally, we verified the effectiveness of each module through experiments, proving the model’s advantages in improving sentiment classification accuracy and robustness.

The model exhibits notable performance degradation on specific sample types, particularly those involving *sarcastic/ironic expressions*or *culture-specific linguistic nuances*. For instance, reviews containing phrases like “Loved waiting 3 hours for cold food!” (paired with generic positive images) or Chinese idioms such as “味同嚼蜡” (“tastes like wax”) in Ctrip data frequently result in misclassification, with F1-scores dropping to 0.62 compared to the overall average of 0.86. This limitation stems from insufficient contextual incongruity detection in the fusion mechanism. Future work will integrate dedicated sarcasm detection modules leveraging contextual incongruity features [9] and culture-specific sentiment lexicons to address these errors.

Computational constraints remain significant for real-time deployment, particularly on edge devices. The current implementation requires 4.4GB of GPU memory (Table 6) and processes 100 samples in 34 seconds using an NVIDIA V100 GPU, exceeding industry standards for real-time sentiment analysis (<1 second per batch). To mitigate this, we propose adopting model quantization (FP16 to INT8) to reduce memory usage by ~40% and deploying distilled architectures (e.g., TinyBERT) to accelerate inference by 2.1 × , thereby enabling resource-efficient deployment in mobile tourism applications.

Performance is highly sensitive to input data quality, with accuracy decreasing by 12.3% when processing low-resolution images (<480p) or machine-translated reviews (e.g., Google-translated English-to-Chinese text in Ctrip data), compared to a < 3% drop in ResNet-only baselines. This vulnerability arises from the attention mechanism’s over-reliance on high-quality multimodal alignment. Planned improvements include robustness layers with adversarial noise injection during training and fine-tuning multilingual BERT on back-translated datasets to enhance resilience to noisy inputs.

Compared to six state-of-the-art multimodal sentiment analysis models, the experimental results of this study indicate that the multimodal sentiment analysis model can effectively combine various data modalities to improve sentiment classification accuracy and reliability.
